# Structuring of Bacterioplankton Diversity in a Large Tropical Bay

**DOI:** 10.1371/journal.pone.0031408

**Published:** 2012-02-21

**Authors:** Gustavo B. Gregoracci, Juliana R. Nascimento, Anderson S. Cabral, Rodolfo Paranhos, Jean L. Valentin, Cristiane C. Thompson, Fabiano L. Thompson

**Affiliations:** 1 Laboratory of Microbiology, Institute of Biology, Federal University of Rio de Janeiro (UFRJ), Rio de Janeiro, Brazil; 2 Laboratory of Hydrobiology, Institute of Biology, Federal University of Rio de Janeiro (UFRJ), Rio de Janeiro, Brazil; 3 Laboratory of Zooplankton, Institute of Biology, Federal University of Rio de Janeiro (UFRJ), Rio de Janeiro, Brazil; 4 Laboratory of Molecular Genetics of Microorganisms, IOC-FIOCRUZ, Rio de Janeiro, Brazil; Argonne National Laboratory, United States of America

## Abstract

Structuring of bacterioplanktonic populations and factors that determine the structuring of specific niche partitions have been demonstrated only for a limited number of colder water environments. In order to better understand the physical chemical and biological parameters that may influence bacterioplankton diversity and abundance, we examined their productivity, abundance and diversity in the second largest Brazilian tropical bay (Guanabara Bay, GB), as well as seawater physical chemical and biological parameters of GB. The inner bay location with higher nutrient input favored higher microbial (including vibrio) growth. Metagenomic analysis revealed a predominance of *Gammaproteobacteria* in this location, while GB locations with lower nutrient concentration favored *Alphaproteobacteria* and *Flavobacteria*. According to the subsystems (SEED) functional analysis, GB has a distinctive metabolic signature, comprising a higher number of sequences in the metabolism of phosphorus and aromatic compounds and a lower number of sequences in the photosynthesis subsystem. The apparent phosphorus limitation appears to influence the GB metagenomic signature of the three locations. Phosphorus is also one of the main factors determining changes in the abundance of planktonic vibrios, suggesting that nutrient limitation can be observed at community (metagenomic) and population levels (total prokaryote and vibrio counts).

## Introduction

Microbes are recognized as major drivers of nutrient cycling in the Oceans and in coastal waters [1]. They present structured populations both in large and small scale patterns [2], [3], [4] and may even share some biogeographical and macroecological features of macroorganisms, according to their geographic distribution [5]. Microbial community diversity analysis in the North Pacific Subtropical gyre indicated the contribution of different microbial taxa to nutrient cycling according to the depth [2]. Microbial diversity may also be structured according to latitude [3] and even within the same location [4], [6]. A global survey of the bacterioplankton community diversity indicated a latitudinal gradient in species richness, with an increasing number of taxa (species) at lower latitudes, and only 2 cosmopolitan taxa out of 562, suggesting a pattern of geographic-dependent microbial diversity structuring [7]. Coastal bacterioplankton and vibrioplankton communities appear to be finely structured in discrete phylogenetic clusters, revealing the co-occurrence of several hundred closely related microbial populations [6]. Sympatric differentiation may be due to niche partitioning and specialization with the association of different groups of bacterioplanktonic species with different habitats (zooplankton, particles, or water) in the same geographic location [4], [6]. Hunt et al. [4] showed that some vibrio species appeared to occur only in association with plankton, whereas other species appear to be exclusively free-living. A few studies have addressed the main water quality parameters that determine structuring shifts in bacterioplanktonic and vibrioplanktonic diversity and abundance [8], [9], [10], [11], [12]. Salinity, phosphorus, and nitrogen concentration appeared to influence the abundance of vibrios in cold water environments [8], [9], [10], [11], [12]. *Vibrio splendidus* and *V. anguillarum* species groups were among the most abundant taxa in cold water environments [9]. Similar studies have not been carried out yet in tropical environments. Thus it is not known if these previous findings can be generalized for other (tropical) environments.

The environmental parameters and ecological factors involved in habitat and/or niche partitioning may comprise biotic and abiotic factors. Three levels of control over microbial abundance and diversity are recognized [13], [14], [15]: i. bottom-up control, or nutrient supply/limitation; ii. top-down control via viral lysis and/or protist grazing, and iii. sideways control, or interactions among co-occurring microbial populations. The interplay in and among these three levels explain community structure and composition, according to niche partitioning theories [1], [16]. On the other hand, experimental studies demonstrated recently that microbial diversity resulting from niche partitioning leads to more efficient nutrient uptake [15], [17], also implying that biodiversity may influence water quality and be advantageous as a buffer against pollution impacts [17]. Since vibrios comprise several human (e.g. *V. cholerae*, *V. vulnificus*, and *V. parahaemolyticus*) and marine animal (e.g. *V. harveyi* species group) pathogenic species [18], [19], it is very important to determine specific environmental triggers for their growth and diversity structuring in tropical environments.

We studied the bacterioplankton abundance and diversity of Guanabara Bay (GB) as a model to better understand the effects of water quality parameters on free-living bacterioplankton, in three representative locations of the GB, according to previous studies [20]. GB is the second largest bay of Brazil and is located near Rio de Janeiro city, one of the largest metropolitan areas of South America, with nearly 9 million people living in close vicinity [21]. Monthly counts of vibrio CFUs and total prokaryotic (bacteria+archaea) counts, prokaryotic production measurements, and measurements of water quality parameters (nutrient concentration, chlorophyll a, salinity, temperature, and pH) allowed us to establish correlations between different parameters. The 24-month time series study (between February 2009 and March 2011) allowed us to investigate the role of nutrient input on the abundance and diversity of total bacterioplankton and vibrioplankton, determining which nutrients limit their growth. Vibrio counts and vibrio diversity were used as proxies for the determination of correlations with water quality parameters because these *Gammaproteobacteria* are known to respond swiftly to nutrient inputs [22]. Additionally, metagenomic analysis of the different sites corroborates some of the inferences from the chemical analysis and resulted in the determination of a metagenomic signature for the GB.

## Materials and Methods

### Study area

**Figure 1:**
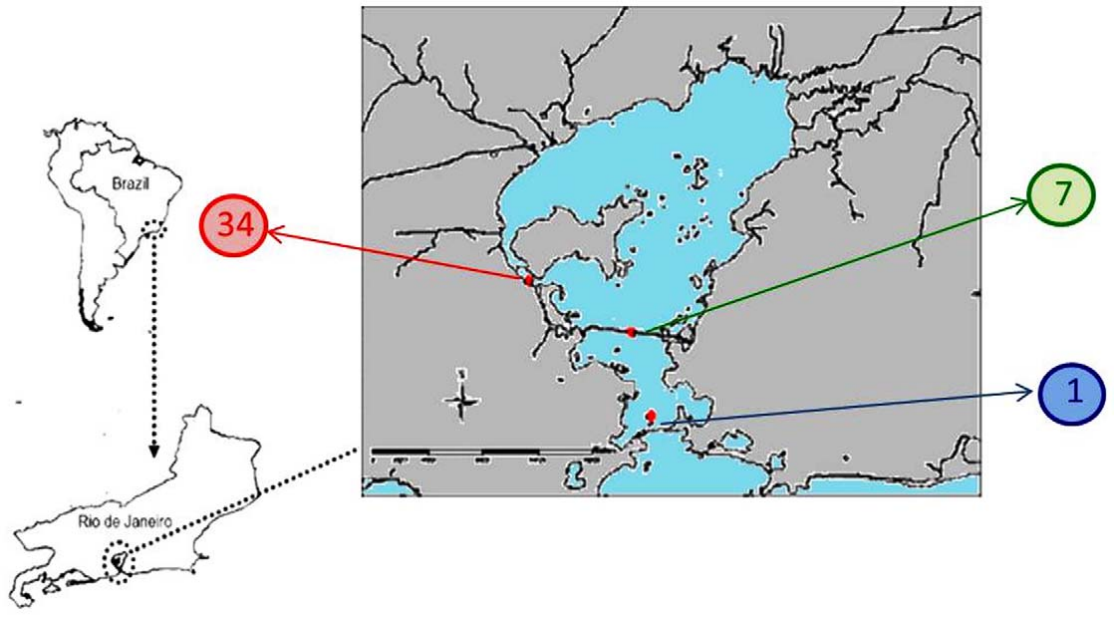
Study site. The Guanabara Bay, our study site, situated in the context of state (Rio de Janeiro) and country (Brazil). Locations marked as 1, 7 and 34 represent the sampling points within the bay (coordinates in the text).

The study area is located in the Guanabara Bay (centered on lat. 22°50′S and long. 43°10′W), Rio de Janeiro State, a eutrophic estuarine system near one of the largest metropolitan areas in Brazil. No specific permits were required for the described field studies. Subsurface waters (1–2 m depth) were sampled from three different sites, representing different trophic levels (Figure 1), according to the literature [20]. Location 1 is at the most external point of the GB (22°55′43″S; 43°08′51″W), therefore receiving high influence of the marine environment and lesser influx of terrigenous material. The second sampling site (location 7) is located in an intermediary point in the middle of the GB (22°52′12″S; 43°09′46″W), subjected to strong mixing of inner and coastal seawater, and the third sampling location (34) is situated near land (22°50′09″S; 43°14′56″W), impacted by anthropogenic activities. Monthly samples were collected between February 2009 to March 2011, and subjected to physical-chemical and microbiological analyses. Microbial production was limited to February 2009 to June 2009. Citometry counts were limited to 2009 only.

### Physical-chemical and biological analyses

All environmental parameters were analyzed by standard oceanographic methods [23]. At least three replicates were analyzed for each parameter monthly. Temperature and salinity were evaluated by using CTD or salinity meters from YSI. Chlorophyll *a* analyses were performed after vacuum filtration (<25 cm of Hg) of 2L of water. The filters (Glass fiber Millipore AP15) were extracted overnight in 90% acetone at 4°C, and analyzed by spectrophotometry or fluorimetry. Inorganic nutrients were also analyzed: 1) ammonia by indophenol, 2) nitrite by diazotization, 3) nitrate by reduction in Cd-Cu column followed by diazotation, 4) total nitrogen by digestion with potassium persulfate following nitrate determination, 5) orthophosphate by reaction with ascorbic acid, 6) total phosphorous by acid digestion to phosphate, and 7) silicate by reaction with molibdate. Dissolved (DOC) and particulate (POC) organic carbon were analyzed as described previously [24].

#### Prokaryotic abundance and productivity

Prokaryotic production and total prokaryotic counts were performed only in the first year of study. Abundance was determined from two replicates of seawater by flow cytometry with Syto-13 (Life Technologies, Carlsbad, CA), with minor modifications [25]. Microbial cells with high (HNA) and low (LNA) nucleic acids content were quantified through measurement of fluorescence intensity by flow cytometry with Syto-13. Prokaryotic production was determined according to Smith and Azam [26], using 3H-leucine incorporation into proteins as a proxy of production. Carbon production was calculated using the conversion factor of 0.86 [27].

#### Vibrios CFU counts and diversity

Monthly seawater samples were serially diluted and plated in triplicate (100 µl) in Thiosulphate Citrate Bile Salts Sucrose (TCBS) agar. Colony counts were performed 48 h after incubation at 28°C. All vibrio colonies obtained in February 2010 from the three locations were stored in Tryptone Soy Broth with 20% glycerol at −80°C. These colonies were purified and isolates were subjected to DNA extraction following [28] and their uridylate kinase (*pyrH*) gene sequences were obtained in order to determine vibrio diversity. PCR amplification of the *pyrH* gene was performed as described previously [29], using primers 80F and 530R. PCR products of the approximate size (approx. 500 bp) were purified through Illustra GFX™ PCR DNA and Gel Band Purification kit, following the manufacturer protocol. Purified PCR products were analyzed in an automatic Applied Biosystems Genetic Analyzer 3500. *pyrH* sequences were analyzed using the software Kodon package 2.03, and compared to reference sequences (type strains) available from GenBank (NCBI). Phylogenetic tree was constructed based on *pyr*H gene sequences applying the Neighbor-joining method using the software MEGA [30]. Distance estimation was obtained by the model of Kimura 2-Parameter. Bootstrap analysis was performed after 2,000 replications. Phylogenetic trees were constructed using the software MEGA [30].

#### Statistical analysis

Simple correlations were initially used as exploratory analysis, through fit of data points in linear models. Total prokaryotic abundance and Vibrio counts were plotted against all other variables and fit to the linear model (r^2^) were calculated in each case, in Microsoft Excel (Microsoft®). Variable transformation was used to ensure normality wherever needed (usually counts vs. abiotic variables). Two additional multivariate analyses were performed using the STATISTICA (Statsoft®) software: a) an ordination analysis, the principal component analysis (PCA), performed on a correlation matrix between total prokaryotic abundance, HNA, LNA, Vibrios and abiotic variables, and b) a multiple regression analysis with total prokaryotic abundance, HNA, LNA and vibrio counts as dependent variables and abiotic parameters as independent variables.

For the multiple regression analysis, assumptions were respected through residual analysis. To avoid distortions in the statistical tests, outlier data (>2*sigma) were excluded and independence between explicative variables was assumed for a *tolerance* >0.01. The *tolerance* of a variable is defined as 1 minus the squared multiple correlation of this variable with all other independent variables in the regression equation. Therefore, the smaller the *tolerance* of a variable, the more redundant is its contribution to the regression (i.e., it is redundant with the contribution of other independent variables).

#### Metagenomic analysis

A deeper understanding of structuring and metabolic potential of the sampled sites involved metagenomic analysis. Two replicate (ca. 2L each) seawater samples collected in February 2010, from each of the three study locations. In total, six samples were pre-filtered in a 20 µm nylon filter, followed by a Sterivex 0.2 µm filter. Microbial cells retained in the Sterivex filter received SET buffer and were stored at −80°C until DNA extraction, which was performed as described previously [31]. Metagenomic DNA samples were sequenced by 454 pyrosequencing [32]. Metagenomic sequences were annotated using MG-RAST 2.0, utilizing the subsystems technology for metabolic analyses and the SEED database for phylogenetic analyses, with an e-value cutoff of 10^−5^ [33]. The main phylogenetic groups were further extracted after SEED analysis, and resubmitted to evaluate their relative contribution to the functional subsystems. The Statistical analysis of metagenomic profiles (STAMP) bioinformatics software was used for statistical analysis [34]. Statistical significance was calculated using two-sided Fisher's exact test, and the differences between proportions were analyzed using the Newcombe-Wilson method with 99% confidence interval. Data was further subjected to filtering and only data with p-values lower than 0.01 were analyzed. Functional data was additionally compared to a pool of 19 public metagenomes from different bays around the world in order to determine possible unique features of GB in comparison to other bays. The list of these bays is available in the Table S1. The analysis was restricted to the sequences only, given that metadata of these bays was not available with the metagenomic sets.

## Results

### Physical chemical analysis

Seawater parameters were measured monthly in three locations of the GB (Table 1). The highest values of inorganic and organic nutrients were observed in location 34, the inner portion of the GB. For instance, total N and total P were 574.36±493.15 µM and 19.43±5.31 µM in location 34, respectively, with ammonia and orthophosphate as main contributors (Table 1). The values of the same parameters in location 1 were 67.73±34.55 µM and 2.69±1.03 µM, respectively. The higher silicate values observed in the location 34 (80.79±70.55 µM) in comparison with location 1 (23.63±13.94 µM) and location 7 (33.57±16.10 µM) suggested a higher contribution of land runoff and/or benthic-pelagic effects. In addition, salinity, pH, seawater transparency, and dissolved oxygen values were lower in the location 34. There were also higher chlorophyll *a* levels and lower pheophytin (chlorophyll degradation product) levels in this location, indicating active photosynthesis. Based on the physical chemical and biological parameters of the seawater, the three locations can be split in two groups: Group 1, corresponding to locations 1 and 7, represented an intermediate area of the GB with higher influence from the coastal waters; Group 2, corresponding to location 34, a heavily polluted area of the GB, and with considerable input of nutrients.

**Table 1:** Average values plus standard deviation of physical chemical water parameters, according to location in GB.

	Site 1	Site 7	Site 34
Microbial counts (cell.ml^−1^)	5,17E6±1,18E6	7,22E6±1,24E6	2,39E7±3,16E6
High Nucleic Acid cells (% of total)	2,9E6±7,42E5 (56)	3,86E6±1,02E6 (53)	1,8E7±2,65E6 (67)
Low Nucleic Acid cells (% of total)	2,72E6±5,82E5 (52)	3,44E6±7,82E5 (47)	9,21E6±1,34E6 (34)
Vibrio count (CFU.ml^−1^)	171,53±164,19	199,27±262,68	4508,15±4825,66
Chlorophyll (µM)	30,84±29,47	46,29±42,63	171,58±143,96
Pheophytin (µM)	5,94±5,27	10,88±10,89	20,68±27,94
Dissolved Organic Carbon (µM)	5,69±2,13	7,62±5,12	9,37±2,59
Ammonium (µM)	10,24±7,39	20,03±20,40	200,83±105,46
Nitrite (µM)	1,79±1,22	2,62±2,32	2,51±1,43
Nitrate (µM)	4,64±8,61	7,68±2,12	15,29±1,62
Total Inorganic Nitrogen (µM)	13,02±11,01	24,94±30,28	223,05±122,13
Total Nitrogen (µM)	67,73±34,55	79,94±39,88	574,36±493,15
Ortophosphate (µM)	1,10±0,46	1,92±1,38	13,02±3,88
Total P (µM)	2,69±1,03	4,09±2,22	19,43±5,31
Total N/Total P	31,12±8,98	26,54±8,98	38,58±28,12
Dissolved Oxygen (ml.L^−1^)	3,38±0,95	3,87±1,43	1,98±2,27
%Saturation of Dissolved Oxygen	72,17±9,10	87,33±17,98	45,97±43,95
Bacterial Production	1,27E3±5,41E2	1,3E3±5,29E2	5,31E3±5E2
Water Temperature	23,50±2,28	24,00±2,67	25,52±2,82
Salinity	31,38±2,82	29,29±3,51	22,07±4,92
pH	8,32±0,11	8,37±0,17	7,92±0,23
Transparency (m)	2,18±1,30	1,43±0,90	0,62±0,36
Silicate (µM)	23,63±13,94	33,57±16,10	80,79±70,55
Suspended particulate matter	44,83±15,85	45,50±11,73	58,94±32,07

### Total prokaryotic counts and vibrio (CFUs) counts

**Figure 2:**
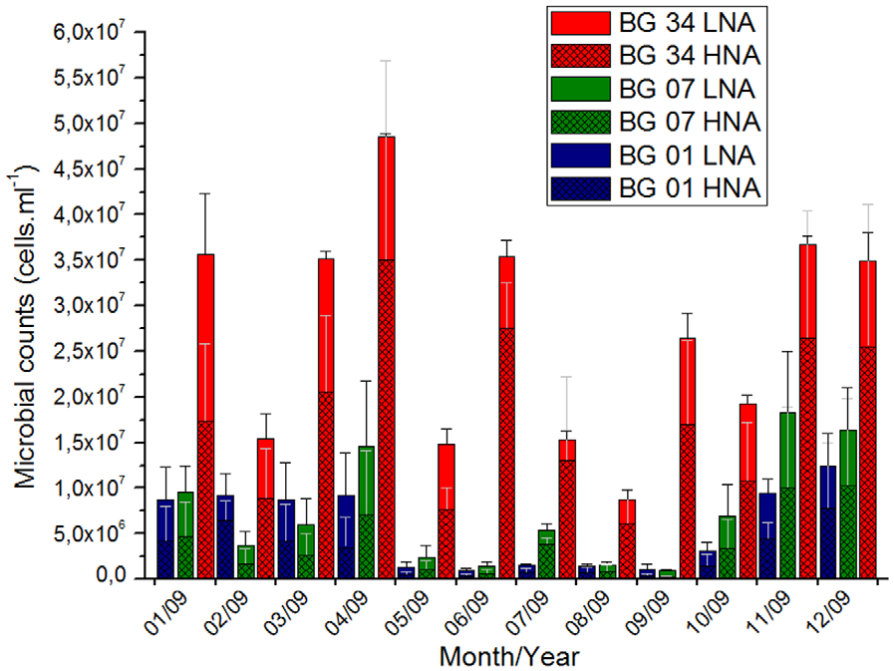
Microbial counts determined through flow citometry. Total count is further divided in cells with high (HNA) and low (LNA) nucleic acid content, as determined through differences in emission of fluorescence. The values represent averages, plus SE.

**Figure 3:**
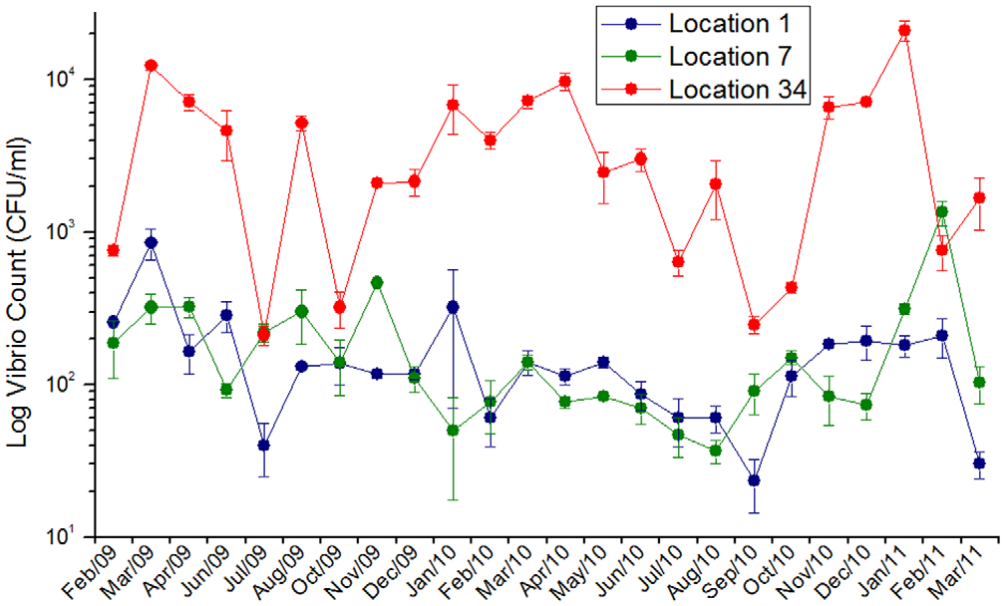
Monthly Vibrio counts determined through TCBS plating. Individual values represented averages, plus SE.

Total prokaryotic counts obtained by flow cytometry and vibrios CFU counts obtained by plating on TCBS were higher in location 34 than in the other two locations (Table 1). Prokaryotic cell counts varied between 9,66×10^5^ and 3,68×10^7^, with the highest values occurring in April 2009 (Figure 2). Vibrios CFU counts varied between 30 and 20,866 CFU/ml, with the highest values occurring in January 2011 (Figure 3). A presumptive seasonal pattern for the microbial abundance was observed, with higher counts in the summer months (Dec–Apr) and lower counts in the winter months (Jul–Sept) (Figure 2 and 3). Prokaryotic cells with high nucleic acid content (HNA) were more abundant in location 34 (representing almost 70% of total counts). On the other hand, the contribution of HNA prokaryotic cells was equal to the contribution of LNA prokaryotic cells in locations 1 and 7, which suggests a lower proportion of dividing cells (Figure 2 and Table 1) [1]. Our results indicated a strong correlation between total prokaryotic counts/prokaryotic production and vibrios CFU counts. The great majority of the vibrio isolates belonged to three species (*Vibrio communis*, *V. parahaemolyticus* and *V. alginolyticus*) according to *pyrH* gene sequence analysis (Figure S1). The *pyrH* sequence diversity (allelic diversity) was higher in locations 1 and 7 than in location 34 (results not shown).

### Relationship between seawater parameters and microbial abundances

The initial exploratory analysis revealed high fits to linear models between total microbial abundance and vibrio counts (r^2^ = 0.829), and between both these counts and microbial production (0.70<r^2^<0.76) (Supplementary Figure S2). Vibrio abundance also fitted the models when plotted against orthophosphate (r^2^ = 0.620) and ammonia (r^2^ = 0.443) showing direct correlation, as opposed to an inverse correlation when plotted against salinity (r^2^ = 0.511) (Figure S2).

**Figure 4:**
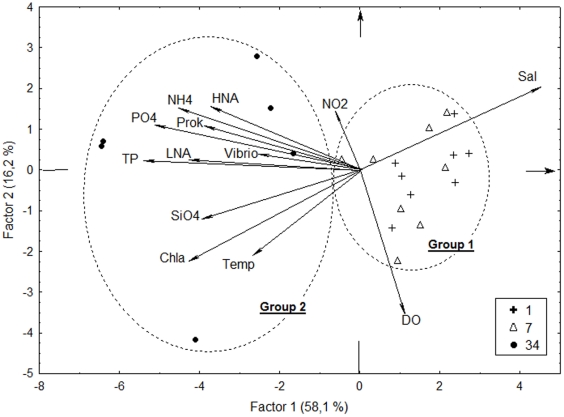
PCA analysis of biotic and abiotic variables. There is a clear division between Group 1 (locations 1 and 7) and Group 2 (location34). Prok: total prokaryotic abundance. HNA: high nucleic acid content cells. LNA: low nucleic acid content cells. Vibrio: Vibrio counts. NH4: ammonia. PO4: orthophosphate. NO2: nitrite. TP: total phosphorus. SiO4: silicate. Chla: chlorophyll *a*. Temp: temperature. Sal: salinity.

The PCA supported the division between Group 1 (locations 1 and 7) and Group 2 (location 34) (Figure 4). All biotic variables clustered with phosphorus (total and orthophosphate) and ammonia, and opposed to salinity, agreeing with the exploratory correlations even though the latter refer only to Vibrio (Figure S2).

The more robust multiple regression analysis also highlighted the importance of ammonia and mainly phosphorus to all biotic variables (Table 2). Total prokaryotic abundance increase was highly dependent (p≤0.011) on increases of orthophosphate, ammonia and temperature, as well as dependent on chlorophyll-*a* decreases (p = 0.043). HNA cells responded to increases in orthophosphate and ammonia (0.036<p<0.038). LNA, on the other hand, was highly statistically dependent only to temperature (p = 0.0006), even though total phosphorus, chlorophyll-*a* and ammonia were also selected for the model with no statistical support (p>0.05). Vibrio abundance was directly dependent on orthophosphate (p = 0.012) and inversely dependent on salinity (p = 0.032), despite nitrite being also selected for the model, with no statistically significant contribution (p = 0.161).

**Table 2:** Multiple regression models through stepwise addition of independent environmental variables.

Multiple Regression Models	Independent variables	Regression coefficient β	Partial correlation	p-value
**Dependent variable: Prokaryotic abundance, R^2^ = 0.791, p<0.0001, n = 26**	Ortophosphate	0.540	0.523	0.010
	Ammonia	0.474	0.518	0.011
	Temperature	0.348	0.539	0.008
	Chlorophyll-*a*	−0.330	−0.426	0.043
**Dependent variable: HNA, R^2^ = 0.714, p<0.0001, n = 23**	Ortophosphate	0.454	0.449	0.036
	Ammonia	0.447	0.444	0.038
**Dependent variable: LNA, R^2^ = 0.666, p<0.0001, n = 23**	Temperature	0.654	0.702	0.0006
	Total phosphorus	0.592	0.429	0.059
	Chlorophyll-*a*	−0.460	−0.439	0.053
	Ammonia	0.225	0.230	0.328
**Dependent variable: Vibrio count, R^2^ = 0.350, p<0.0001, n = 62**	Ortophosphate	0.361	0.321	0.012
	Salinity	−0.310	−0.277	0.032
	Nitrite	−0.15	−0.183	0.161

### Metagenomic analysis description

To better analyze the structure of the bacterioplankton and to further assess the metabolic potential of the microbes, a total of six microbial plankton samples were subjected to metagenomic analysis. Location 1 produced 72,907 and 96,780 sequences, from which 85,723 (50.52%) were classified in the MG-RAST subsystems hierarchy and 112,245 (66.15%) were classified in the SEED database (Table S2). Location 7 produced 10,848 and 59,432 sequences, with 37,306 (53.08%) classified in the MG-RAST metabolic subsystems and 45,999 (65.45%) in the SEED database. Location 34 produced 14,883 and 6,581 sequences, from which 11,548 (53.82%) were classified in the MG-RAST metabolic subsystems and 14,869 (69.30%) in the SEED database.

### Taxonomic assignment of the GB metagenomes

**Figure 5:**
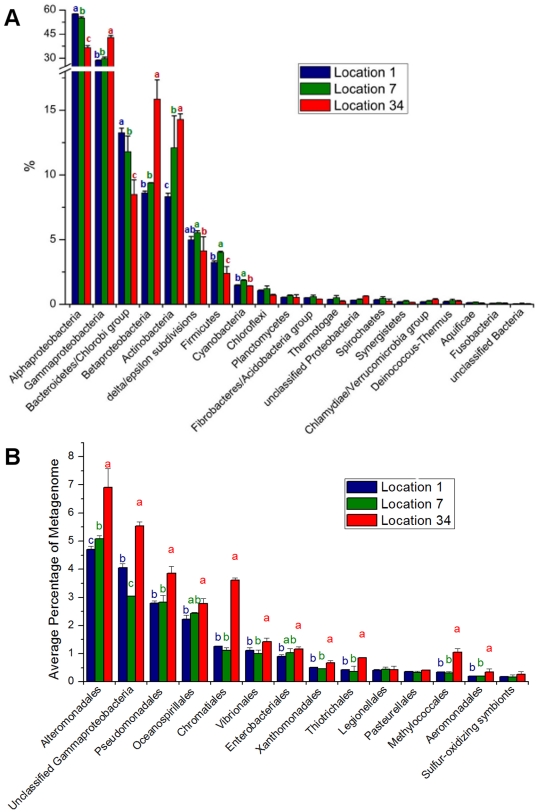
Relative percentage of contribution of phylogenetic groups to metagenomes, separated by locations. Different letters indicate significant difference (p<0,01) between samples, while repeated letters indicate no statistical difference. In all cases, *a*>*b*>*c*, regarding relative percentage values. **A**) Major bacterial groups present in BG. *Proteobacteria*, the most abundant group, was further subdivided into main classes. **B**) Major groups within the class *Gammaproteobacteria*.

The relative majority of sequences recovered from GB belonged to the domain *Bacteria*. Archaeal sequences corresponded to less than 1% of the total, mainly represented by methane metabolizing groups (Figure S3). Around 1% of the sequences belonged to eukaryotes and viruses (Figure S4). Phages and large algal viruses (but not prophages) were more abundant in location 7, suggesting higher ongoing bacterial infection of these groups, since most of them should have been filtered out during sampling instead of remaining in the microbial fraction (however see [35]). There was a high relative abundance of *Proteobacteria* in the three locations, corresponding to around 60–68% of all metagenomic sequences (Figure 5A). A shift was observed from *Alphaproteobacteria* and *Bacteroidetes/Chlorobi* in locations 1 and 7 to *Gammaproteobacteria, Betaproteobacteria* and *Actinobacteria* in location 34. *Flavobacteria*, *Rhodobacterales* and *Pelagibacter ubique* were the main contributors to this pattern of relative abundance shift observed in locations 1 and 7 (Figure S5), while *Actinobacteridae*, *Burkholderiales* and several groups within *Gammaproteobacteria* (*Alteromonadales*, unclassified *Gammaproteobacteria*, *Pseudomonadales*, *Chromatiales*, *Vibrionales* and a few others) were responsible for the higher relative abundance in the more polluted location 34 (Figure 5B).

### Predominant metabolic profiles of the GB

**Figure 6:**
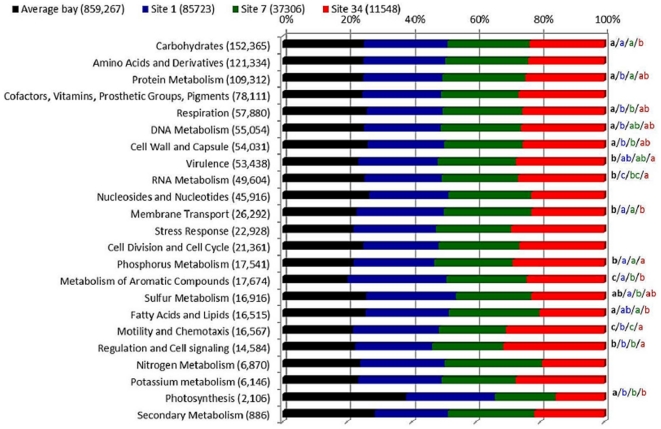
MG-RAST metabolic subsystems. Metabolic profiles of GB locations and the average bay metagenome (pooled data from 19 public metagenomes). All subsystems are normalized to 100%. Letters on the right side of the bars indicate significant differences (p<0,01) between samples, while repeated letters indicate no statistical difference. In all cases, *a*>*b*>*c*, regarding relative percentage values.

Metagenomic sequences of the three locations were classified in 23 informative subsystems (Figure 6). Locations 1 and 7 had relatively more metagenomic sequences related to carbohydrates and membrane transport subsystems, while location 34 had more metagenomic sequences related to RNA metabolism, virulence, motility and chemotaxis, regulation and cell signaling, cell division and cell cycle subsystems. When compared with other bay systems around the world, GB appeared to have some singular metabolic features (Figure 6). GB had proportionally less sequences in the photosynthesis subsystem and more sequences in the phosphorus metabolism and metabolism of aromatic compounds than the mean profile of 19 pooled bay metagenomes (descriptions provided in Table S1). Location 34 represents an outlier, in comparison with the average bay and the other locations, showing a lower proportion of carbohydrates and fatty acids subsystems and higher RNA metabolism, virulence, motility/chemotaxis and regulation/cell signaling. Location 34 was more similar to the average bay metagenome than to locations 1 and 7 only in a few subsystems. There was a lower relative number of sequences within the membrane transport subsystem in both location 34 and the average bay metabolic profile, than in the locations 1 and 7. On the other hand, GB metagenomic sequences identified in the respiration, cell wall/capsule, and cell division/cell cycle subsystems were more abundant in location 34 and the average bay metabolic profile than in locations 1 and 7.

### Relationship between metabolic (subsystems) and taxonomic information

**Figure 7:**
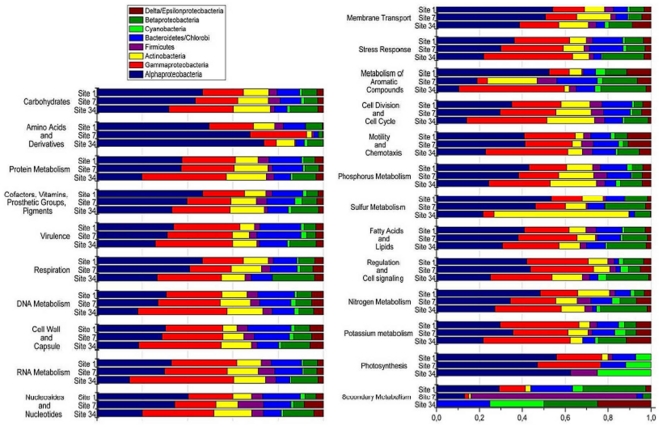
Relative contribution to metabolic subsystems of the main phylogenetic groups present in the GB. All subsystems are normalized to 100%.

*Alphaproteobacteria* predominated in locations 1 and 7 and were reduced in the polluted location 34. *Gammaproteobacteria*, on the other hand, predominated in location 34 and were reduced in the locations 1 and 7. These relative shifts were reflected in the contribution of these groups to all subsystems in each location (Figure 7). For instance, *Gammaproteobacteria* had a higher contribution to several subsystems (such as phosphorous metabolism) in location 34. An increased contribution of *Betaproteobacteria* sequences to several subsystems was also noted in location 34, while the opposite was observed in *Bacteroidetes/Chlorobi* group, also reflecting changes in abundance in these groups. *Cyanobacteria* had a higher proportional contribution to the photosynthesis subsystem in the location 34, despite the similar relative abundances observed. *Actinobacteria* was also proportionally more abundant in location 34 and contributed predominantly to sulfur metabolism in this site (Figure 7).

## Discussion

Physical, chemical and biological parameters of GB seawater reflected two groups of environments (Table 1 and Figure 4). Locations 1 and 7 suffered more influence of oceanic seawater, in contrast with location 34. Even though previous spatial studies suggested differences between locations 1 and 7 [20], our time-series sampling revealed highly similar environments, corroborating the notion that location 7 is prone to water mixing, with greater influence of oceanic water. The concentration of some nutrients in the inner location of the GB was well above other coastal systems. For instance, ammonia and phosphate concentration in location 34 of GB were, respectively, 6.8 and 6 times the highest average value in 26 years of measurement in the Delaware Bay [36]. GB ammonia and phosphate levels were also about 7 and 20 times, respectively, the values obtained in the Yangtze estuary [37]. Total N and total P were, respectively, 9 and 5 times higher in location 34 of the GB than in the riverine station of the Neuse estuary (US) [38]. The relatively low pH, dissolved organic carbon, and dissolved oxygen in location 34 clearly indicated intense consumption of nutrients and high biochemical oxygen demand. The high loads of nutrients in the inner portion of the GB appear to have a drastic influence in microbial metabolism, abundance and diversity.

### Bottom-up effects as main regulators of bacterioplankton and vibrioplankton abundance and diversity in the GB

The multiple regression models covered most, but not all, of the variation (0.666<model r^2^<0.791), suggesting that other controlling factors, such as predation by protists and viruses, may account for prokaryotic abundance variation in GB, particularly in the vibrio model (r^2^ = 0.350). Future studies are needed in order to evaluate their ecological role in (vibrio) abundance and diversity in GB. Nevertheless, linear model fits revealed phosphorus (orthophosphate and total phosphorus) as one of the main limiting factors for vibrio growth in the GB, and the more robust multiple regression models supported this view for total prokaryotic abundance, HNA and vibrios, but not for LNA cells (Table 2). LNA cell abundance increase was highly dependent on temperature increases (p = 0.0006), hinting at the influence of temperature on cell division activation, at least in this tropical bay. Phosphorus appeared to be an important factor in previous studies in the Swedish coastline and the Western English Channel [8], [9], [10], [11]). On the other hand, the effect of phosphorus on the vibrioplankton of the Chesapeake Bay was not evident, since temperature, dissolved-O_2_ concentration, and tide height yielded the highest correlations with vibrio abundance [12].

The N∶P ratios observed in GB (31∶1, 26∶1 and 38∶1 in locations 1, 7 and 34, respectively) and the higher prevalence of phosphorus metabolism subsystem in metagenomes from all locations supports the idea of phosphorus limitation. Tropical estuarine systems are usually phosphorus limited, even though in general, coastal systems are limited by nitrogen [39]. This occurs because land runoff generally supplies phosphorus to coastal systems [39], unlike open ocean waters [2]. In spite of the high loads of phosphorus that GB receives, it appears that the even higher carbon and nitrogen input from anthropogenic sources (particularly ammonia) supply the demand for carbon and nitrogen, allowing larger population size which is, in turn, limited by phosphorus. In addition, prokaryotes may need to compete for phosphorus with other fractions of the plankton, such as photosynthesizing eukaryotes, which appeared to be abundant near location 34, which is supported by the dependence of total prokaryotic abundance to the decrease in chlorophyll-*a* observed in the multiple regression model. Salinity also influenced the abundance of vibrios and may account for a fraction of microbial diversity variation as suggested previously [8], [9], [10], [11].

### High nutrient loads promote microbial growth and affect microbial composition and diversity in GB

A clear relationship between nutrient concentration and microbial abundance was observed. Total microbial counts were at least one order of magnitude higher in location 34 than in locations 1 and 7. In addition, a higher proportion of actively dividing microbial cells (suggested by higher HNA counts as discussed in Kirchman [1]) was observed in location 34. On the other hand, the excessive input of nutrients may also have reduced the overall diversity of the GB, as observed in other aquatic systems [17]. A reduction in the estimated number of bacterial species was observed in location 34 (407 OTUs) in comparison to locations 1 (455 OTUs) and 7 (488 OTUs). The vibrio *pyrH* allelic diversity was also lower in location 34 (results not shown). The metagenomic analysis allowed some inferences regarding the structuring of the microbial populations. *Gammaproteobacteria*, *Betaproteobacteria* and *Actinobacteria* were proportionally the most abundant groups in location 34. The contribution of the former two to the metabolism in this location were generally higher in most subsystems, while *Actinobacteria* seemed to explore a particular niche metabolizing sulfur compounds (Figure 7). This finding needs to be explored further in future studies. In contrast, the more oligotrophic locations 1 and 7 presented lower microbial counts and nutrient concentrations and selected for different bacterioplanktonic groups, particularly *Alphaproteobacteria* and *Flavobacteria*. These two groups had a proportionally higher contribution to most metabolic subsystems (Figure 7).

### The GB has a distinct metagenomic signature

Because most subsystems (e.g. amino acids and derivatives, protein metabolism, cofactors, and phosphorus metabolism) were found to be equally abundant regardless of the sample location (1, 7, or 34) and the taxonomic group, it appears that a selection of microbes adapted to the GB environmental conditions occurred. Overall, GB had a distinct metabolic profile compared to an average bay metagenome (pooled data from 19 public bay metagenomes obtained from MG-RAST). Phosphorus metabolism represented one of the distinctive features in the metagenomes and was more (relative) abundant in GB than in other bays. Indeed, phosphorus seemed to be a limiting factor in GB due to excessive C and N input. Metagenomic sequences belonging to the aromatic compounds subsystems were also proportionally more abundant in the GB than in other bays. Aromatic compounds are persistent pollutants that accumulate in the water because they are hard to digest [40] and may represent a nutrient source, particularly valuable in the more oligotrophic locations 1 and 7. This is compatible with a notion that the GB is chronically polluted with hydrocarbons [41]. The photosynthesis subsystem was poorly represented in the microbial (prokaryotic) metabolic profile of GB. The high concentrations of chlorophyll-*a* observed in the inner location 34, with proportionally lower pheophytin concentrations indicates that primary producers were abundant in the GB, but such organisms compose larger fractions of the (eukaryotic)plankton, not analyzed in this study. Because the GB had a distinct metagenomic profile, we suggest that metagenomic signatures may reflect environmental effects on microbial populations of GB, supporting the view that metagenomic signatures are useful to describe environmental features [42]. Although a more detailed analysis accounting for environmental factors of each bay would be preferred, the lack of metadata accompanying these public metagenomes prevented such approach. The different coverage should not prevent our tentative conclusions given that the analysis was restricted to the top-level hierarchy of MG-RAST, and that the trends observed were common to all six GB metagenomes (irrespective of their sizes).

### Vibrios can be considered indicators of trophic conditions

Vibrios are indigenous marine bacteria, which grow easily on plates and play important ecologic roles in the marine environment [29]. They are also known for their swift responses to nutrient rich environments, with some species having duplication times as low as 10 minutes in suitable conditions [22], [43]. Because some vibrio species are human and/or animal pathogens, it may be important to monitor vibrio counts in coastal waters. Promoting the growth of super-heterotrophic potentially pathogenic bacteria may lead to disease in marine life, such as benthic invertebrates and corals [44], [45], [46]. The abundance of vibrios in GB was one order of magnitude higher in location 34 than locations 1 and 7, similar to total microbial counts. Significant simple correlations were observed between vibrios counts and both total microbial counts and microbial production. As such, vibrios counts seems a good proxy (indicator organism) for water quality, as suggested in Dinsdale et al. [46], and we propose that vibrios counts of >200 CFU/mL might reflect polluted seawaters (inference from Figure 3). A potential health risk is highlighted here, since GB waters are used for recreation (swimming, bathing, fishing), and *Vibrio parahaemolyticus* was among the most frequently found species in these waters. Additionally, *Vibrio* plating is a relatively inexpensive technique for monitoring bacterial numbers in the environment, unlike the more expensive and technically demanding flow citometry count.

This is the first study on the Guanabara Bay aiming at a time series comprehensive analysis on the planktonic microbial diversity and abundance. Our approach comprised nutrient concentration measurements, microbial counts, and metagenomics, to unravel microbial population composition and metabolic potential. The integration of data allowed us to shed light on the main bottom up factors controlling microbial abundance in GB. Our data also shows that the GB has typical features (e.g. phosphorus metabolism) that differentiate it from other bay metagenomic signatures. These features may have been acquired in the course of the occupation of the GB area, leading to in the observed structuring of the microbial composition. We also show that nutrient (phosphorus) limitation may be inferred from community (metagenome) and population (total prokaryote and vibrio) levels.

## Supporting Information

Figure S1 **Phylogenetic tree based on pyrH gene sequences of vibrio isolates from Guanabara Bay using the Neighbor-joining method.** Distance estimation was obtained by the model of Kimura 2-Parameter. Bootstrap percentages after 2,000 replications are shown. Scale bar, 1% estimated sequence divergence. Evolutionary analyses were conducted in MEGA5.
(TIF)

Figure S2 **Most meaningful fits to linear models between vibrio/microbial counts and physical chemical parameters.** Data points from each location are marked differently according to the legend. An exponential fit (data log transformed) was deemed best in figures B, C, D, E, F, G and H to ensure normality.
(TIF)

Figure S3 **Relative percentage of contribution of archaeal sequences to GB metagenomes, separated by locations.** Different letters indicate significant difference (p<0,01) between samples, while repeated letters indicate no statistical difference. In all cases, *a*>*b*>*c*, regarding relative percentage values.
(TIF)

Figure S4 **Relative percentage of contribution of viral sequences to metagenomes, separated by locations.** Different letters indicate significant difference (p<0,01) between samples, while repeated letters indicate no statistical difference. In all cases, *a*>*b*>*c*, regarding relative percentage values.
(TIF)

Figure S5 **Relative percentage of contribution of alphaproteobacterial sequences to metagenomes, separated by locations.** Different letters indicate significant difference (p<0,01) between samples, while repeated letters indicate no statistical difference. In all cases, *a*>*b*>*c*, regarding relative percentage values.
(TIF)

Table S1 **List of public Bay metagenomes and general data obtained from MG-RAST.**
(XLSX)

Table S2 **General data regarding GB metagenomes.**
(XLSX)

## References

[1] Kirchman DL (2008). Microbial ecology of the oceans. 2nd ed. ed.

[2] DeLong EF, Preston CM, Mincer T, Rich V, Hallam SJ (2006). Community genomics among stratified microbial assemblages in the ocean's interior.. Science.

[3] Fuhrman JA, Steele JA, Hewson I, Schwalbach MS, Brown MV (2008). A latitudinal diversity gradient in planktonic marine bacteria.. Proceedings of the National Academy of Sciences of the United States of America.

[4] Hunt DE, David LA, Gevers D, Preheim SP, Alm EJ (2008). Resource partitioning and sympatric differentiation among closely related bacterioplankton.. Science.

[5] Nemergut DR, Costello EK, Hamady M, Lozupone C, Jiang L (2011). Global patterns in the biogeography of bacterial taxa.. Environmental microbiology.

[6] Acinas SG, Klepac-Ceraj V, Hunt DE, Pharino C, Ceraj I (2004). Fine-scale phylogenetic architecture of a complex bacterial community.. Nature.

[7] Pommier T, Canback B, Riemann L, Bostrom KH, Simu K (2007). Global patterns of diversity and community structure in marine bacterioplankton.. Molecular ecology.

[8] Gilbert JA, Field D, Swift P, Thomas S, Cummings D (2010). The taxonomic and functional diversity of microbes at a temperate coastal site: a ‘multi-omic’ study of seasonal and diel temporal variation.. PloS one.

[9] Eiler A, Johansson M, Bertilsson S (2006). Environmental influences on Vibrio populations in northern temperate and boreal coastal waters (Baltic and Skagerrak Seas).. Applied and environmental microbiology.

[10] Lozupone CA, Knight R (2007). Global patterns in bacterial diversity.. Proceedings of the National Academy of Sciences of the United States of America.

[11] Gilbert JA, Field D, Swift P, Newbold L, Oliver A (2009). The seasonal structure of microbial communities in the Western English Channel.. Environmental microbiology.

[12] Louis VR, Russek-Cohen E, Choopun N, Rivera IN, Gangle B (2003). Predictability of Vibrio cholerae in Chesapeake Bay.. Applied and environmental microbiology.

[13] Azam F, Fenche T, Field JG, Gray JS, Meyer-Reil LA (1983). The Ecological Role of Water-Column Microbes in the Sea.. Marine Ecology - Progress Series.

[14] Fuhrman JA (1999). Marine viruses and their biogeochemical and ecological effects.. Nature.

[15] Gravel D, Bell T, Barbera C, Bouvier T, Pommier T (2011). Experimental niche evolution alters the strength of the diversity-productivity relationship.. Nature.

[16] Leibold MA (2008). Ecology: Return of the niche.. Nature.

[17] Cardinale BJ (2011). Biodiversity improves water quality through niche partitioning.. Nature.

[18] Austin B, Austin D, Sutherland R, Thompson F, Swings J (2005). Pathogenicity of vibrios to rainbow trout (Oncorhynchus mykiss, Walbaum) and Artemia nauplii.. Environmental microbiology.

[19] Thompson FL, Iida T, Swings J (2004). Biodiversity of vibrios.. Microbiol Mol Biol Rev.

[20] Vieira RP, Gonzalez AM, Cardoso AM, Oliveira DN, Albano RM (2008). Relationships between bacterial diversity and environmental variables in a tropical marine environment, Rio de Janeiro.. Environmental microbiology.

[21] IBGE (2010).

[22] Eilers H, Pernthaler J, Amann R (2000). Succession of pelagic marine bacteria during enrichment: a close look at cultivation-induced shifts.. Applied and environmental microbiology.

[23] Grasshoff K, Kremling K, Erhardt M (1999). Methods of seawater analysis.

[24] Rezende CE, Pfeiffer WC, Martinelli LA, Tsamakis E, Hedges JI (2010). Lignin phenols used to infer organic matter sources to Sepetiba Bay - RJ, Brasil.. Estuarine Coastal and Shelf Science.

[25] Andrade L, Gonzalez AM, Araujo FV, Paranhos R (2003). Flow cytometry assessment of bacterioplankton in tropical marine environments.. Journal of Microbiological Methods.

[26] Smith DC, Azam F (1992). A simple, economical method for measuring bacterial protein synthesis rates in seawater using ^3^H-leucine.. Marine Microbial Food Web.

[27] Simon M, Azam F (1989). Protein content and protein synthesis rates of planktonic marine bacteria.. Marine Ecology Progress Series.

[28] Pitcher DG, Saunders NA, Owen RJ (1989). Rapid Extraction of Bacterial Genomic DNA with Guanidium Thiocyanate.. Letters in Applied Microbiology.

[29] Thompson FL, Gevers D, Thompson CC, Dawyndt P, Naser S (2005). Phylogeny and molecular identification of vibrios on the basis of multilocus sequence analysis.. Applied and environmental microbiology.

[30] Tamura K, Dudley J, Nei M, Kumar S (2007). MEGA4: Molecular Evolutionary Genetics Analysis (MEGA) software version 4.0.. Molecular biology and evolution.

[31] Thompson FL, Bruce T, Gonzalez A, Cardoso A, Clementino M (2011). Coastal bacterioplankton community diversity along a latitudinal gradient in Latin America by means of V6 tag pyrosequencing.. Archives of microbiology.

[32] Margulies M, Egholm M, Altman WE, Attiya S, Bader JS (2005). Genome sequencing in microfabricated high-density picolitre reactors.. Nature.

[33] Meyer F, Paarmann D, D'Souza M, Olson R, Glass EM (2008). The metagenomics RAST server - a public resource for the automatic phylogenetic and functional analysis of metagenomes.. BMC bioinformatics.

[34] Parks DH, Beiko RG (2010). Identifying biologically relevant differences between metagenomic communities.. Bioinformatics.

[35] Fuhrman JA, Liang X, Noble RT (2005). Rapid detection of enteroviruses in small volumes of natural waters by real-time quantitative reverse transcriptase PCR.. Applied and environmental microbiology.

[36] Yoshiyama K, Sharp JH (2006). Phytoplankton Response to Nutrient Enrichment in an Urbanized Estuary: Apparent Inhibition of Primary Production by Overeutrophication.. Limnology and Oceanography.

[37] Nianzhi J, Zhao Y, Luo T, Wang X (2006). Natural and anthropogenic forcing on the dynamics of virioplankton in the Yangtze river estuary.. Journal of the Marine Biological Association of the United Kingdom.

[38] Burkholder JM, Dickey DA, Kinder CA, Reed RE, Mallin MA (2006). Comprehensive Trend Analysis of Nutrients and Related Variables in a Large Eutrophic Estuary: A Decadal Study of Anthropogenic and Climatic Influences.. Limnology and Oceanography.

[39] Ferguson Wood EJ (1967). Microbiology of Oceans and Estuaries.

[40] Seo JS, Keum YS, Li QX (2009). Bacterial degradation of aromatic compounds.. International journal of environmental research and public health.

[41] Christensen JH, Tomasi G, de Lemos Scofield A, de Fatima Guadalupe Meniconi M (2010). A novel approach for characterization of polycyclic aromatic hydrocarbon (PAH) pollution patterns in sediments from Guanabara Bay, Rio de Janeiro, Brazil.. Environmental pollution.

[42] Dinsdale EA, Edwards RA, Hall D, Angly F, Breitbart M (2008). Functional metagenomic profiling of nine biomes.. Nature.

[43] Aiyar SE, Gaal T, Gourse RL (2002). rRNA promoter activity in the fast-growing bacterium Vibrio natriegens.. Journal of bacteriology.

[44] Dinsdale EA, Rohwer F, Dubinsky Z, Stambler N (2011). Fish or Germs? Microbial Dynamics Associated with Changing Trophic Structures on Coral Reefs.. Coral Reefs: An Ecosystem in Transition: Springer Netherlands.

[45] Vezzulli L, Previati M, Pruzzo C, Marchese A, Bourne DG (2010). Vibrio infections triggering mass mortality events in a warming Mediterranean Sea.. Environmental microbiology.

[46] Dinsdale EA, Pantos O, Smriga S, Edwards RA, Angly F (2008). Microbial ecology of four coral atolls in the Northern Line Islands.. PloS one.

