# Supplementary figures and images for: Structuring of Bacterioplankton Diversity in a Large Tropical Bay
Source: PLoS One. 2012 Feb 21;7(2):e31408. doi: 10.1371/journal.pone.0031408 (PMC3283626; doi:10.1371/journal.pone.0031408)

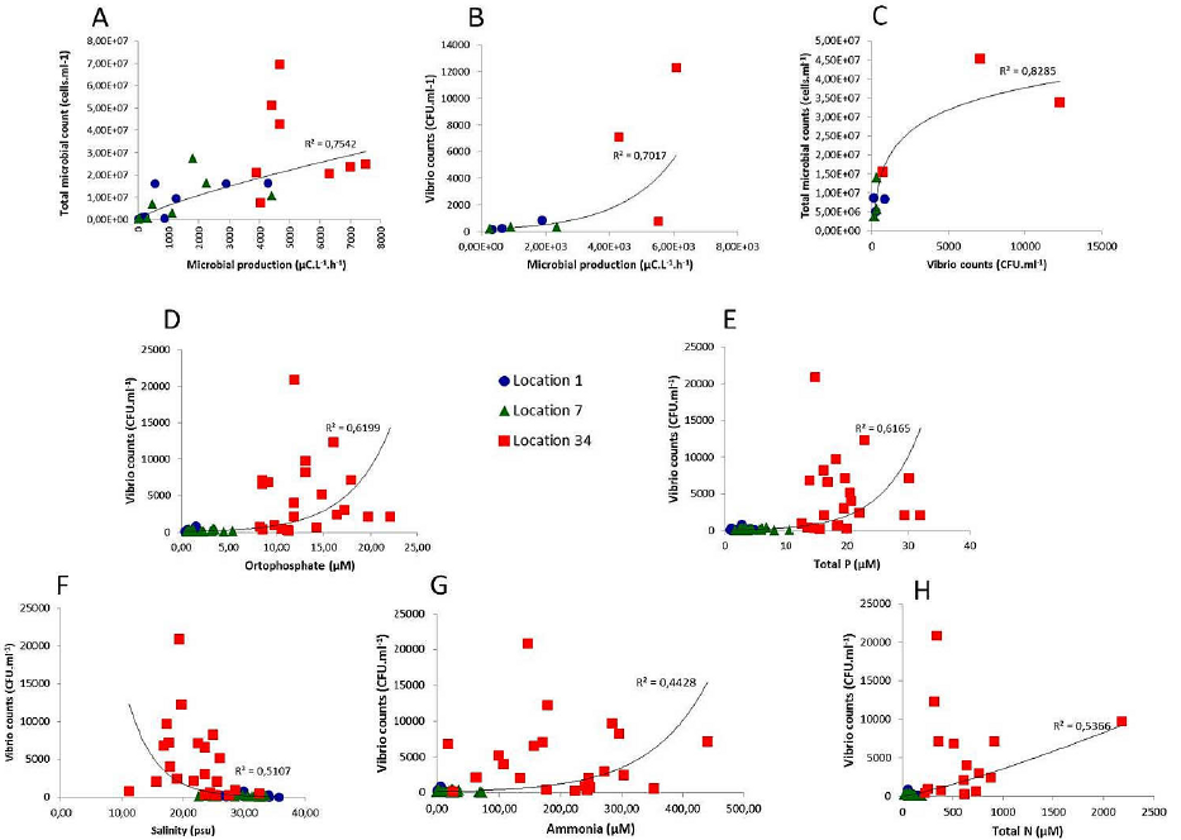

Supplement: Figure S2 — Most meaningful fits to linear models between vibrio/microbial counts and physical chemical parameters. Data points from each location are marked differently according to the legend. An exponential fit (data log transformed) was deemed best in figures B, C, D, E, F, G and H to ensure normality. (TIF) [file pone.0031408.s002.tif]

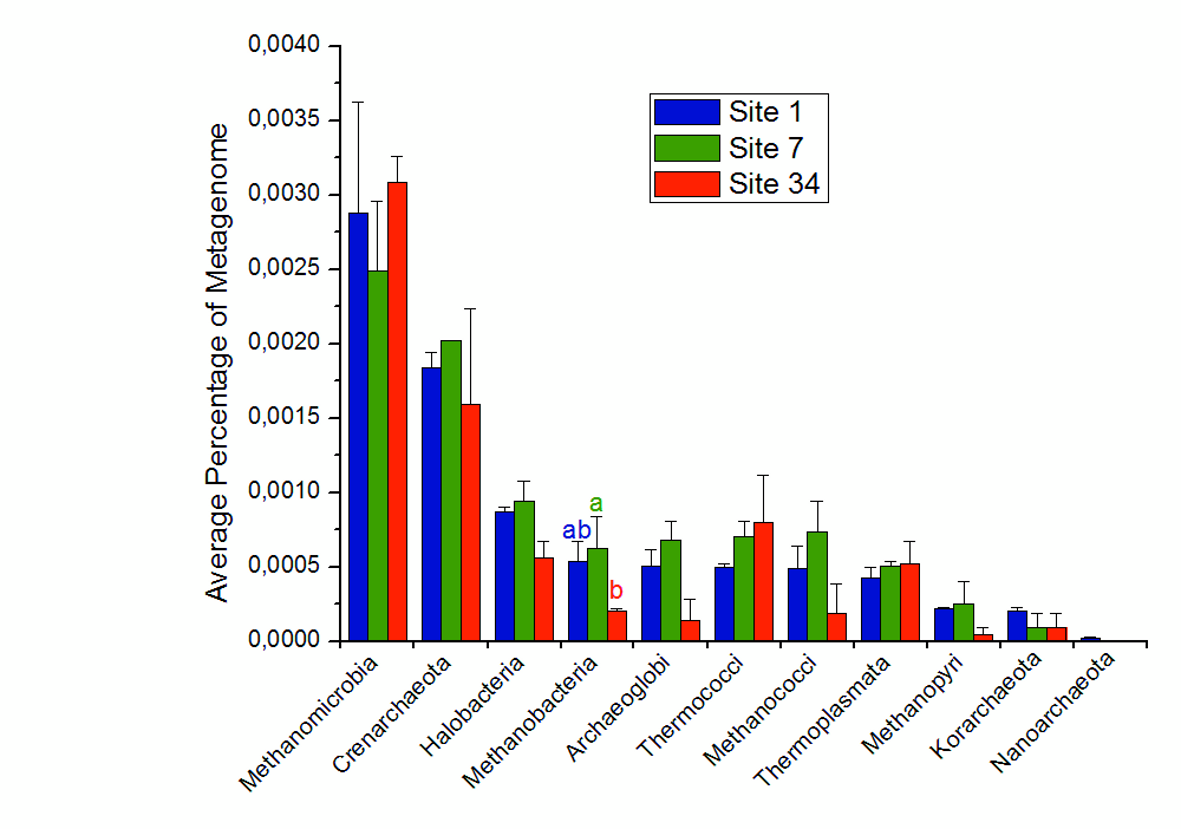

Supplement: Figure S3 — Relative percentage of contribution of archaeal sequences to GB metagenomes, separated by locations. Different letters indicate significant difference (p<0,01) between samples, while repeated letters indicate no statistical difference. In all cases, a>b>c, regarding relative percentage values. (TIF) [file pone.0031408.s003.tif]

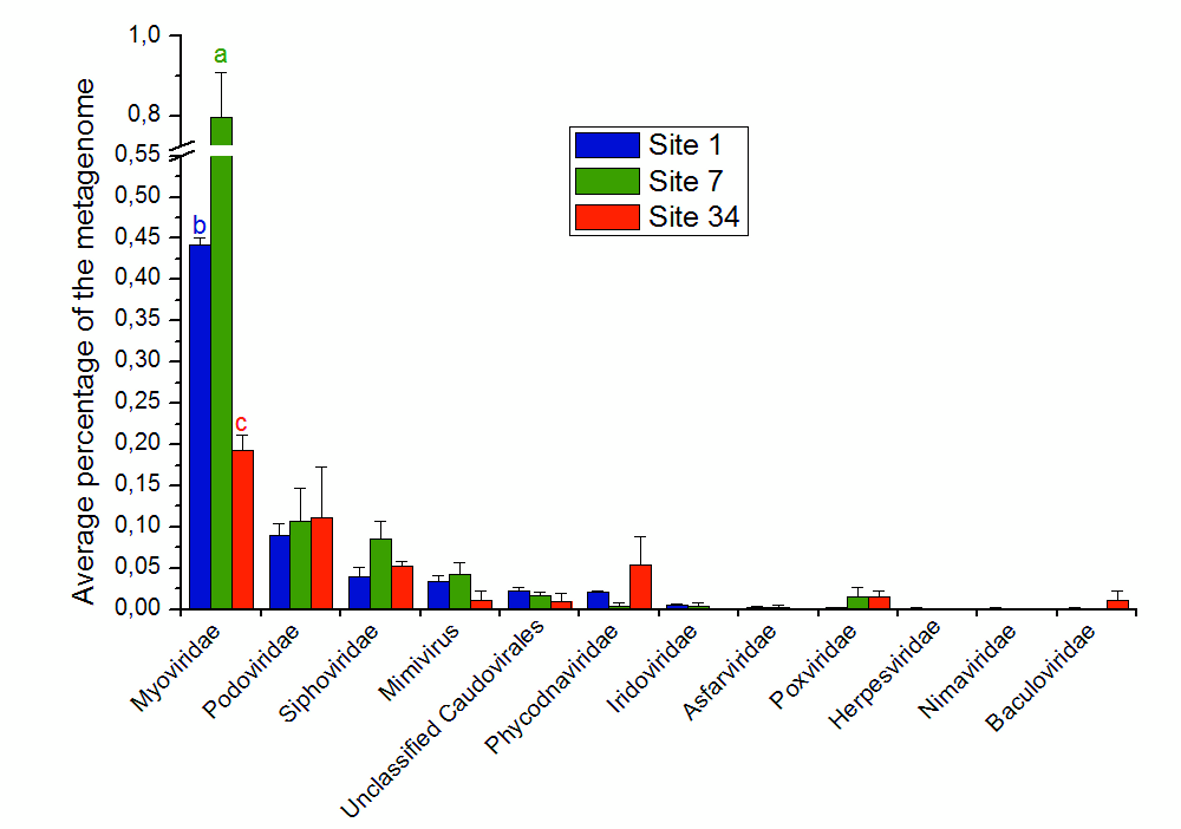

Supplement: Figure S4 — Relative percentage of contribution of viral sequences to metagenomes, separated by locations. Different letters indicate significant difference (p<0,01) between samples, while repeated letters indicate no statistical difference. In all cases, a>b>c, regarding relative percentage values. (TIF) [file pone.0031408.s004.tif]

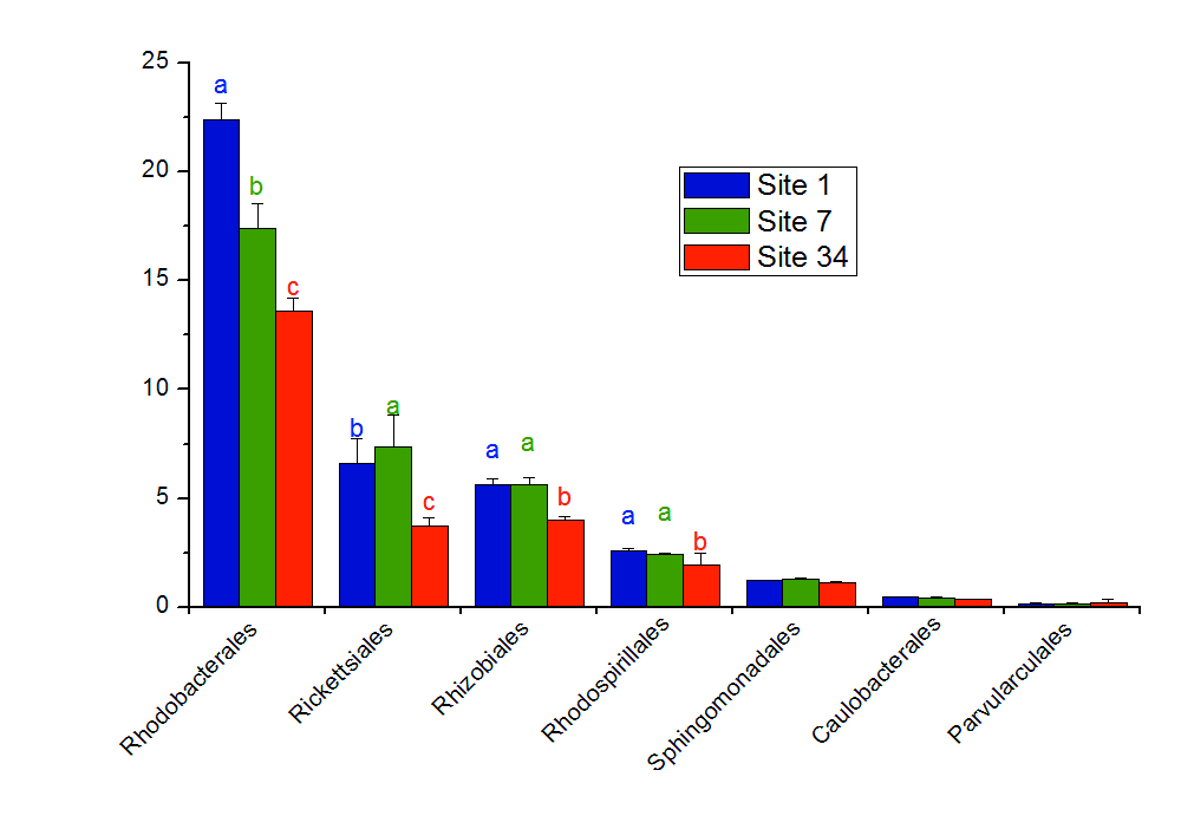

Supplement: Figure S5 — Relative percentage of contribution of alphaproteobacterial sequences to metagenomes, separated by locations. Different letters indicate significant difference (p<0,01) between samples, while repeated letters indicate no statistical difference. In all cases, a>b>c, regarding relative percentage values. (TIF) [file pone.0031408.s005.tif]
